# Buried Alive

**Published:** 1872-10

**Authors:** 


### BURIED ALIVE.

THIS is a comparatively rare event. If a needle is stuck deep into the flesh, it comes out tarnished, if there is life; if none, then it is as bright as before it was used.

